# Probiotics: A Dietary Factor to Modulate the Gut Microbiome, Host Immune System, and Gut–Brain Interaction

**DOI:** 10.3390/microorganisms8091401

**Published:** 2020-09-11

**Authors:** Tetsuji Hori, Kazunori Matsuda, Kenji Oishi

**Affiliations:** Yakult Central Institute, 5-11 Izumi, Kunitachi-shi, Tokyo 186-8650, Japan; tetsuji-hori@yakult.co.jp (T.H.); kazunori-matsuda@yakult.co.jp (K.M.)

**Keywords:** probiotics, gut microbiome, immune control, gut–brain interaction, health

## Abstract

Various benefits of probiotics to the host have been shown in numerous human clinical trials. These organisms have been proposed to act by improving the balance of the gut microbiota and enhancing the production of short-chain fatty acids, as well as by interacting with host cells in the gastrointestinal tract, including immune cells, nerve cells, and endocrine cells. Although the stimulation of host cells by probiotics and subsequent signaling have been explained by in vitro experiments and animal studies, there has been some skepticism as to whether probiotics can actually interact with host cells in the human gastrointestinal tract, where miscellaneous indigenous bacteria coexist. Most recently, it has been shown that the ileal microbiota in humans after consumption of a fermented milk is occupied by probiotics for several hours, indicating that there is adequate opportunity for the ingested strain to stimulate the host cells continuously over a period of time. As the dynamics of ingested probiotics in the human gastrointestinal tract become clearer, further progress in this research area is expected to elucidate their behavior within the tract, as well as the mechanism of their physiological effects on the host.

## 1. Introduction

Diet is one of the factors affecting human health status. For example, excess sugars intake is linked to the development and exacerbation of diabetes and obesity, and excess lipid intake is linked to hyperlipidemia and cardiovascular disorders. In contrast, moderate dietary fiber intake promotes the production of short-chain fatty acids (SCFAs) by the intestinal microbiota, resulting in improved bowel movements and a reduced risk of infection and obesity [1]. Probiotics are considered to be dietary factors that can influence human health.

Recently, probiotics, as represented by lactobacilli and bifidobacteria, have become a familiar part of our dietary habits and are available in various forms, including fermented milk, tablets, biscuits, and chocolates. Probiotics have been defined as “live microorganisms which when administered in adequate amounts confer a health benefit on the host” by the Food and Agriculture Organization and World Health Organization [2], and as “a live microbial food ingredient that, when ingested in sufficient quantities, confers health benefits on the consumer” by the International Life Science Institute [3]. As is clear from these definitions, the requirements for probiotics are to be alive and to confer a beneficial effect on the host. Before these definitions were made, “confer beneficial effect on the host” was explained as “by improving its intestinal microbial balance” [4]. In fact, there have been some reports of the physiological effects linked to changes in the host gut microbiota [5,6,7,8,9]. On the other hand, a different type of mechanism in which probiotics exert their physiological effects by interacting directly with host cells has also been discussed. The human gut microbiota, so far, has generally been investigated by using fecal microbiota analyses. Very recently, changes in the human ileal microbiota after the ingestion of probiotic fermented milk have been shown by the analysis of ileal fluids [10], and the dynamics of probiotics within the gastrointestinal tract are now becoming clearer.

In this review, we outline the relationship between the influence of probiotic intake on the gut microbiota and the physiological effects of these bacteria on the host, and we present the latest findings on immune control and gut–brain interaction, as exerted through the direct interactions of probiotics with host cells.

## 2. Effect on Gut Microbiota

This chapter describes the changes in gut microbiota induced by probiotic intake and the relationship between these changes and their physiological effects on the host.

### 2.1. Neonates

Gut microbiota of vaginally delivered neonates are greatly affected by inoculation with the microorganisms in the mother’s vagina and anorectum [11,12], whereas those of cesarean section newborns are strongly affected by inoculation with environment-derived microorganisms [13]. Yang et al. [14] categorized 26 Chinese neonates into three groups and analyzed the effects of probiotic intake during lactation on the process of gut microbiota formation. Nine neonates born by cesarean section, compared with three vaginally delivered neonates, had significantly lower α-diversity of fecal microbiota at 28 days of age. In each of seven neonates born by cesarean section and fed a total of 10^9^ colony-forming units (CFUs) or 10^10^ CFUs of *Bifidobacterium lactis* Bi-07, *Bifidobacterium lactis* HN019, and *Lactobacillus rhamnosus* HN001 complex, the relative abundances of Lactobacillus and Bifidobacterium in the fecal microbiota increased significantly from 3 days of age, and α-diversity and β-diversity at 28 days of age became comparable to those of vaginally delivered neonates. Bifidobacterial supplementation of maternal colostrum and breast milk in very-preterm infants (VPIs) accelerates the establishment of the bifidobacterial microbiota in their gut [9]. Oshiro et al. allocated 35 VPIs born at between 24 and 31 weeks of gestation and with body weights < 1500 g to two groups and gave either 2.5 × 10^8^ CFUs/day live *Bifidobacterium breve* BBG-01 or placebo for 8 weeks. In comparison with the 18 VPIs in the placebo group, the 17 VPIs given BBG-01 had significantly greater cumulative weight gain by 8 weeks, as well as significantly higher fecal bifidobacterial counts and SCFA concentrations. In that study, more than 80% of the VPIs in both groups were born by cesarean section. During formation of the gut microbiota in the neonatal period, colonization by bifidobacteria is thought to determine future health by “protecting the host from pathogens” and “contributing to the establishment of the mucosal immune system” [15,16,17]. The reports by Yang et al. [14] and Oshiro et al. [9] reveal that administration of probiotics to neonates born by cesarean section may be an important factor promoting the formation of a normal gut microbiota.

Intake of *Lactobacillus rhamnosus* GG by pregnant and postpartum women increases bifidobacterial diversity and the prevalence of *Bifidobacterium longum* species in the fecal microbiota of their infants, thus encouraging the development of a healthy infant-type microbiota [18,19]. Makino et al. [20] isolated identical bifidobacterial strains from the feces of each mother before childbirth and her vaginally delivered neonate after birth; transmission of bifidobacterial strains from the mother thus greatly influenced the formation of the neonatal gut microbiota [12]. On the other hand, administration of *Lactobacillus fermentum* CECT5716, which was isolated from human breast milk, to infants from the age of 6 months to 12 months (a 6-month duration) led to the reduction in the incidence rates of gastrointestinal infections, upper respiratory tract infections, and total number of infections [21]. The transmission of some bacteria presents in the maternal gut to the mammary gland during late pregnancy and lactation through an endogenous route involving maternal dendritic cells and macrophages has been discussed [22]. These suggest that maintenance of a healthy maternal gut microbiota by taking probiotics is an important determinant of the gut microbiota and future health of offspring, and that selected strains isolated from breast milk can be good candidates of probiotics for neonates.

### 2.2. Adults

Changes in adults’ fecal microbiota in response to probiotic intake have also been reported. For example, Ferrario et al. [6], in a randomized, double-blind, crossover placebo-controlled trial, examined the fecal microbiota of healthy adults (23 to 55 years) of both sexes before and after 4 weeks of daily consumption of a capsule containing at least 24 billion viable *Lactobacillus paracasei* DG. Ingestion of the probiotic induced an increase in the relative abundance of Proteobacteria (*p* = 0.006) and the Clostridiales genus Coprococcus (*p* = 0.009) and a decrease in the Clostridiales genus Blautia (*p* = 0.036). A trend of reduction in abundance was also observed in Anaerostipes (*p* = 0.05) and Clostridium (*p* = 0.06). In those subjects with initial fecal butyrate levels > 100 mmol/kg of wet feces, a reduction in butyrate levels of approximately 50% (49% ± 21%) and a concomitant decrease (*p* = 0.021) in abundance of the sum of the six Clostridiales genera (Faecalibacterium, Blautia, Anaerostipes, Pseudobutyrivibrio, Clostridium, and Butyrivibrio) was detected after probiotic intervention. Conversely, in subjects with initial butyrate levels < 25 mmol/kg of wet feces, probiotic intervention induced an approximately three-fold (329% ± 255%) increment in butyrate levels concomitantly with a 55% decrease in Ruminococcus (*p* = 0.016) and a 150% increase in an abundantly represented unclassified Bacteroidales genus (*p* = 0.05). Previous reports indicate that an increase in the Blautia:Coprococcus ratio by ingestion of *Lactobacillus paracasei* DG can potentially confer a health benefit on autistic children [23], HIV-infected subjects [24], and irritable bowel syndrome (IBS) patients [25,26]. However, this probiotic effect on the fecal microbiota and on SCFAs seems to depend strictly on the initial characteristics of the intestinal microbial ecosystem. Ferrario et al. [6] suggested that fecal butyrate concentrations could be an important biomarker for identifying subjects who may benefit from *Lactobacillus paracasei* DG intake.

Chen et al. [27] analyzed the fecal microbiota of 20 healthy men (aged 20 to 25 years) of normal body mass index (BMI) by 16S rRNA gene sequencing after 4 weeks of probiotic intake (2 g containing 1.32 × 10^11^ CFUs live bacteria, including strains of *Lactobacillus acidophilus*, *Lactobacillus rhamnosus* GG, *Bifidobacterium animalis*, and *Bifidobacterium longum*). They reported a significant increase in the relative abundances of *Faecalibacterium prausnitzii* (*p* = 0.043) and Prevotella (*p* = 0.001) compared with those in 20 healthy subjects in the placebo intake group, but there were no obvious differences in α- and β-diversity. Chen et al. [27] also measured serum trimethylamine-*N*-oxide and its precursors, which may contribute to the risk of developing atherosclerosis and cardiovascular disease [28], but they did not detect any attenuation of these parameters associated with detectable changes in the fecal microbiota upon probiotic intake.

The effects of probiotics on the elderly have also been reported. In a placebo-controlled, double-blind study of elderly residents in an aged care facility by Nagata et al. [8], a reduction in the incidence of fever and an improvement in the frequency of defecation were observed in a group (*n* = 36) that received fermented milk containing *Lactobacillus casei* Shirota (at least 4 × 10^10^ CFUs) for 6 months compared with a placebo group (*n* = 36). Concomitantly, the fecal acetate concentration and the numbers of Bifidobacterium and Lactobacillus were significantly higher in the *Lactobacillus casei* Shirota fermented milk intake group than in the placebo group. In contrast, the number of *Clostridium difficile*—a harmful bacterium—was significantly lower in the group that received *Lactobacillus casei* Shirota fermented milk than in the placebo group. Thus, a relationship was observed between the effect of probiotics in protecting against infection with harmful bacteria or improving bowel habits and improvement of the gut microbiota.

### 2.3. Constipation

Multiple studies have consistently demonstrated decreased abundance of bifidobacteria and lactobacilli and increased abundance of Bacteroidetes in patients with constipation, as well as in constipation-predominant IBS [29,30,31,32]. There have been several reports that administration of probiotics to constipated subjects induces changes in the fecal microbiota as well as an improvement in the frequency of constipation. For example, Ishizuka et al. [33] reported an increase in total bifidobacteria after intake of *Bifidobacterium animalis* subsp. *lactis* GCL2505 (10^10^ CFUs/day for 2 weeks), and Matsumoto et al. [5] found that intake of *Lactobacillus casei* Shirota (4 × 10^9^ CFUs/day for 2 weeks) induced an increase in the numbers of Bifidobacterium and total Lactobacillus. On the other hand, Kim et al. [31] reported that administration of VSL#3, which contains three *Bifidobacterium* species, four *Lactobacillus* species, and *Streptococcus thermophiles* (9 × 10^11^ CFUs/day for 2 weeks) did not change the numbers of bifidobacteria or Bacteroides bacteria, although improvements in stool consistency and bowel movements were observed. One of the plausible mechanisms for the improvement of constipation symptoms by taking probiotics is the normalization of intestinal function through increased production of SCFAs in association with balancing of the microbiota [34]. Several human intervention studies have reported changes in the concentrations of fecal SCFAs (i.e., acetate, propionate, and butyrate) in association with the improvement of constipation symptoms [5,35,36]. However, in contrast, in some studies changes in fecal SCFAs were not detected, even though constipation symptoms were improved [37,38]. As more than 95% of SCFAs are absorbed during transit through the colon [39], the concentration in the feces may not accurately reflect the concentration in the colon [40]. In addition, differences in probiotic strains and subjects among trials may have led to these discrepancies in results.

### 2.4. Obesity

The effects of probiotic intake on obese adults have also been reported. The fecal microbiota in obese people is characterized by a high proportion of Firmicutes and a relatively low proportion of Bacteroidetes. In a double-blind placebo-controlled study of 50 obese (BMI > 30 kg/m^2^) youths (12 to 15 years), Larsen et al. [41] reported a significant (*p* < 0.05) increase in the ratio of Bacteroides–Prevotella–Porphyromonas to Firmicutes in the fecal microbiota after treatment with *Lactobacillus salivarius* Ls-33 (10^10^ CFUs/day for 12 weeks) compared with a placebo group. There was no significant change in fecal SCFA concentrations and no improvement in metabolic syndrome, indicating a lack of linkage with changes in the fecal microbiota. Alternatively, a report of the administration of probiotics in combination with fructooligosaccharides and inulin revealed a link between decreased obesity and changes in the intestinal microbiota. In a double-blind, placebo-controlled study by Sanchez et al. [7] of 125 obese men and women (18 to 55 years) (BMI between 29 and 41 kg/m^2^), there was no difference in mean body weight between the placebo group (*n* = 24) and the probiotic group (*n* = 24) in male subjects. In contrast, a significant (*p* = 0.02) reduction in mean body weight was observed in female subjects (*n* = 38) receiving *Lactobacillus rhamnosus* CGMCC1.3724 (3.2 × 10^8^ CFUs with oligofructose and inulin/day for 24 weeks) compared with placebo (*n* = 39). Not only significant decreases in fat mass and circulating blood leptin (a peptide hormone that regulates appetite and energy balance) concentrations, but also a relative decrease in abundance of the Lachnospiraceae family (belonging to the Firmicutes phylum) in the fecal microbiota was detected in those female subjects who lost weight.

## 3. Immune Control

Probiotics affect host immune cells and have positive effects on cancer, infectious diseases such as upper respiratory tract infections (URTIs), allergy, inflammatory bowel disease (IBD) and autoimmune diseases [42,43,44,45,46,47,48,49]. In this section, we focus on their effects on URTIs, IBD, and cancer via host immune control.

### 3.1. URTIs

The immune surveillance systems of children, the elderly, athletes, and stress-burdened workers are relatively weak, making these populations susceptible to URTIs such as the common cold and influenza [50,51,52,53]. Maintaining the immune surveillance system lowers the risk of URTIs. As part of this system, the activity of natural killer (NK) cells, which attack cells infected by viruses and pathogens, is one of the indicators of immunity. Increased NK cell activity is important in URTI prevention [54]. As shown in Table 1, various probiotic strains, such as *Lactobacillus casei* DN-114001, *Lactobacillus delbrueckii* subsp. *bulgaricus* OLL1073R-1; *Lactobacillus casei* Shirota; *Lactobacillus brevis* KB 290; *Bifidobacterium animalis* subsp. *lactis* BB-12; probiotic sachet containing *Bifidobacterium bifidum* W23, *Bifidobacterium lactis* W51, *Enterococcus faecium* W54, *Lactobacillus acidophilus* W22, *Lactobacillus brevis* W63, and *Lactococcus lactis* W58; *Lactobacillus paracasei* N1115; and *Lactobacillus plantarum* DR7 have demonstrated efficacy against URTIs [55,56,57,58,59,60,61,62,63,64].

Makino et al. [55] conducted two independent trials on elderly subjects, allocating to one group of 72 administered yoghurt fermented with *Lactobacillus bulgaricus* OLL1073R-1, while one of 70 was given milk, for at least 8 weeks. Their results showed that the risk of catching a cold was about 2.6 times lower in the yoghurt group than in the milk group, and NK cell activity was significantly higher in the yoghurt group than in the milk group. A randomized controlled study was held of 96 male stress-burdened office workers who were allocated to two groups and given milk as control drink or a fermented milk drink containing 100 billion *Lactobacillus casei* Shirota daily for 12 weeks. The URTI-free rate was 0.78 in the probiotic group but 0.47 in the control group. NK cell activity was significantly higher at week 6 in the probiotic group than in the control group. In contrast to the change in NK cell activity, saliva cortisol—a stress marker—was significantly lower at week 6 in the probiotic group than in the control group [63]. These results suggest that probiotics increase or maintain NK cell activity in the elderly and in stress-burdened office workers and, as a result, could protect from URTIs.

Several probiotics, including *Lactobacillus fermentum* CECT5716 [65], *Bifidobacterium lactis* HN019 [66], *Lactobacillus delbrueckii* subsp. *bulgaricus* OLL1073R-1 [55], *Lactobacillus rhamnosus* HN001 [67], and *Lactobacillus casei* Shirota [63,68,69,70], have been reported to augment NK cell activity. *Lactobacillus casei* Shirota was studied in nine healthy adults with relatively low NK cell activity. *Lactobacillus casei* Shirota fermented milk containing 40 billion CFUs was given daily for 3 weeks. The NK cell activity was significantly augmented in the *Lactobacillus casei* Shirota group after 3 weeks of intake of the probiotic drink; however, 6 weeks after cessation of probiotic intake, the NK cell activity had decreased to the basal level, suggesting that this probiotic can augment NK activity in healthy people with low NK cell activity, but that intake must be regular [68]. In another study, 10 elderly subjects between 69 and 97 years old consumed placebo or a *Lactobacillus casei* Shirota fermented milk containing 40 billion CFUs daily for 3 weeks. Although NK cell activity before ingestion did not differ between the groups, it was significantly increased in the *Lactobacillus casei* Shirota group compared with the placebo group [69].

### 3.2. IBD

IBD is thought to be an aberrant response of mucosal immunity toward the gut microbiome, accompanied by a variety of inflammatory manifestations, including alterations in cytokine production. Ulcerative colitis (UC) and Crohn’s disease are considered to be types of IBD because of their similar symptoms. Although the use of probiotics in IBD and especially UC is still controversial, there have been a few reports that probiotics are beneficial in IBD. *Escherichia coli* Nissle 1917, *Lactobacillus rhamnosus* GG and *Lactobacillus rhamnosus*, *Lactobacillus casei* Shirota, and VSL#3 have had positive effects on IBD in human studies [71,72,73,74,75].

In a randomized controlled study of 187 UC patients, *Lactobacillus rhamnosus* GG did not significantly decrease relapse rates compared with mesalazine (standard treatment), but treatment with *Lactobacillus rhamnosus* GG was more effective than mesalazine in prolonging the relapse-free time (*p* < 0.05) [71]. Moreover, *Lactobacillus rhamnosus* modulated dendritic cell (DC) function, leading to a decrease in T-cell proliferation and interleukin (IL)-2 release. These results suggest that *Lactobacillus rhamnosus* suppresses the overactive immune response in IBD patients and extends the remission period [76]. In terms of the efficacy of probiotic mixtures, Tursi et al. [73] showed that the disease activity index in a group given VSL#3 was significantly (*p* < 0.05) lower than that in the placebo group in a randomized trial. Ng et al. [77] reported that intake of VSL#3 increased IL-10 production and decreased IL-12 levels in the DCs of UC patients, suggesting that this probiotic mixture had anti-inflammatory effects. Mitsuyama et al. [72] studied the effect of a *Lactobacillus casei* Shirota beverage on the abdominal symptoms of 19 patients with UC. The disease activity index in the *Lactobacillus casei* Shirota group was significantly lower than that in the control group. Furthermore, they obtained an interesting insight into the active component of *Lactobacillus casei* Shirota. In an ex vivo study, PSPG-1 (polysaccharide–peptidoglycan complex 1)—a cell-wall component of *Lactobacillus casei* Shirota—significantly inhibited production of the inflammatory cytokine IL-6 in peripheral blood mononuclear cells from UC patients.

### 3.3. Cancer

Some researchers have reported the effects of probiotics on cancer. Changes in immune parameters have not been fully evaluated, but there are reports of effects of probiotics on cancer development. For example, in prospective studies, intake of yoghurt containing *Streptococcus thermophilus* and *Lactobacillus delbrueckii* subsp. *bulgaricus* was inversely associated with colorectal cancer risk [78,79]. Rafter et al. [80] demonstrated reductions in the levels of several cancer biomarkers, along with increased production of interferon gamma (IFN-γ), which is associated with anti-cancer effects, after oral treatment with *Lactobacillus rhamnosus* GG, *Bifidobacterium lactis* Bb12, and inulin in a randomized control trial. Interestingly, *Lactobacillus casei* Shirota has had positive effects against a few cancers, including bladder, breast, and colon cancers [81,82,83,84,85].

Ohashi et al. [82] investigated the effect of *Lactobacillus casei* Shirota-fermented milk on the recurrence of bladder cancer. A significant reduction in the recurrence of bladder cancer was observed in subjects who habitually consumed *Lactobacillus casei* Shirota-fermented milk. Toi et al. [85] conducted an epidemiological study to examine the effect of dietary habits on breast cancer. The participants were 968 Japanese women aged 40 to 55 years. Their lifestyle habits, such as diet and frequency of exercise, from childhood to the time of the study were surveyed. The questionnaire answers were analyzed for factors influencing the incidence of breast cancer. The risk of breast cancer in women with low consumption of *Lactobacillus casei* Shirota fermented milk (fewer than 4 times/week) was set as 1; the relative risk of breast cancer was 0.65 with an increase in the consumption of *Lactobacillus casei* Shirota fermented milk to at least 4 times/week. There was a significant (*p* < 0.05) difference between the two groups. This finding suggests that *Lactobacillus casei* Shirota fermented milk may inhibit the development of breast cancer. Ishikawa et al. [83] investigated whether the consumption of *Lactobacillus casei* Shirota could prevent the occurrence of colorectal tumors. The subjects were 398 men and women aged 40–65 years presently free from tumor who had had at least 2 colorectal tumors removed. There was no significant difference in the development of new colorectal tumors between a control group and a group given a *Lactobacillus casei* Shirota preparation daily for 2 years, but the occurrence rate of tumors with a grade of moderate or higher atypia was significantly decreased by the ingestion of a *Lactobacillus casei* Shirota preparation after 4 years. These results suggested that *Lactobacillus casei* Shirota may slow the progression of colorectal tumors.

## 4. Gut–Brain Interaction

Recent research has revealed that the gut microbiota influences human brain function via gut–brain interaction, and it is likely that ingestion of probiotics helps to maintain and improve mental health. In this section, we introduce the roles of probiotics in human psychological homeostasis involving the neuroendocrine system.

### 4.1. Involvement of Gut Microbiota

The concept of “gut–brain interaction”, which refers to bidirectional communication between the brain and the gut, was first introduced by William James and Carl Lange in the 1880s. Signals from the brain modify the motor, sensory, and secretory modalities of the gastrointestinal tract and, in turn, signals from the gut can affect emotional behavior and stress- and pain-modulation systems. Over the past 15 years, huge amounts of evidence have been presented indicating that the gut microbiota also affect brain function via gut–brain interaction. From this, the concept of “microbiota–gut–brain interaction” evolved. The observation that germ-free (GF) mice, which lack a gut microbiota, show enhanced secretion of stress markers—plasma ACTH (adrenocorticotropic hormone) and corticosterone—when compared with specific-pathogen-free mice (which have a microbiota) under restraint stress [86], was the first indication that the gut microbiota plays important roles in the stress response. The commensal gut microbiota was proven to be an important key factor in development of the central nervous system (CNS), as its colonization process initiates signaling mechanisms that affect neuronal circuits involved in motor control and anxious behavior [87]. Additionally, substantial contributions of the commensal gut microbiota to microglia homeostasis was observed; microglia are tissue macrophages of the CNS and play an important role in repair and maintenance of nerve tissue. GF mice displayed global defects in microglia with altered cell proportions and an immature phenotype, leading to impaired innate immune responses [88]. A study of fecal microbiota transplantation from depressive human patients into GF mice showed that the recipient mice displayed more depression-like behavior than control mice that had received fecal microbiota from healthy subjects, suggesting that the gut microbiota affects mood in mice [89].

In the communication network of the microbiota–gut–brain interaction, the central, autonomic, and enteric nervous systems, the immune system, and the endocrine system are all involved [90,91]. The intestinal microbiota has the potential to affect the nervous system in a direct or indirect manner through bacterial cell components and microbial metabolites such as SCFAs, vitamins, and some types of neurotransmitters [92]. It has been suggested that the microbiota can stimulate enteroendocrine cells (EECs) to release gastrointestinal hormones (e.g., serotonin, ghrelin, peptide YY, glucagon-like peptide 1) and can also stimulate immune cells to release cytokines; signals are then sent to the brain via vagal sensory afferents or via the blood stream [93]. As a consequence, the CNS returns signals to modify the functions of the gastrointestinal tract [92]. A recent study demonstrated that the liver indirectly senses the gut microenvironment, activates the hepatic vagal sensory afferents, and transmits the signals to the brain, so as to modulate gut immune homeostasis through the vagal reflex, including the induction and maintenance of peripheral regulatory T cells [94]. The liver is a key organ that can communicate with many other parts of the human body, and much attention has been paid to its involvement in the microbiota–gut–brain interaction. The mucosal barrier plays an important role in this network, and dysfunction of the mucosal barrier may cause an influx of bioactive luminal components into the body and directly stimulate signal transduction to the brain. It has also been shown that the microbiota can affect the hypothalamic–pituitary–adrenal (HPA) axis through gut–brain interaction [86]. The HPA axis controls biological responses to stress stimuli and is involved in the control of digestion, the immune system, mood and emotional status, sexuality, and energy storage and expenditure. Dysregulation of HPA activity is associated with mental health disorders such as depression and schizophrenia, both of which are known to affect microbiota composition [95].

### 4.2. Modulation of the Microbiota–Gut–Brain Axis by Probiotics

In the context that intestinal microorganisms influence the brain function and psychological state of their host, probiotics have been investigated for their mental health benefits via the gut–brain interaction. Probiotics that confer such benefits are now called psychobiotics, which was originally defined as a live organism that, when ingested in adequate amounts, produces a health benefit in patients suffering from psychiatric illness [96]. Several animal studies have demonstrated that administration of probiotics maintains mucosal barrier function under stressful situations [97,98] and mitigates stress-induced glucocorticoid and inflammatory cytokine responses; this mitigation is accompanied by a reduction in depression- and anxiety-related behavior [98,99,100,101,102,103]. Moreover, probiotics reduce the expression of receptors for the inhibitory neurotransmitter GABA (γ-aminobutyric acid) and the expression of cFos, a marker of neuronal activity, in the brain [101,103], possibly by modulating the gut–brain axis [100,101].

Several human trials have demonstrated the function of probiotics in controlling anxiety and depression. Benton et al. [104] conducted a randomized controlled trial in healthy subjects consisting mainly of aged adults (*n* = 132, aged 48 to 79 years) to examine the effects of *Lactobacillus casei* Shirota (6.5 × 10^9^ CFUs/day for 3 weeks) on mood and cognitive function. In a subgroup with a high depressive index at baseline, intervention subjects showed a significant improvement in depressive mood compared with subjects who received a placebo control (*p* < 0.04). This observation was supported by the results of randomized, double-blind, placebo-controlled studies targeting petrochemical workers [105] and patients with IBS [106]. The study by Mohammadi et al. [105] demonstrated that administration of probiotic yoghurt or capsules containing *Lactobacillus acidophilus* LA5 and *Bifidobacterium lactis* BB12 for 6 weeks improved mental health parameters of petrochemical workers (*n* = 70), as measured by a general health questionnaire and a depression anxiety and stress scale. In another study, by Pinto-Sanchez et al. [106], the effects of *Bifidobacterium longum* NCC3001 (1 g of probiotic powder containing 1.0 × 10^10^ CFUs/day for 6 weeks) on anxiety and depression in patients with IBS (*n* = 44) were evaluated. There, more patients given probiotic powder had reduction in depression scores than those given placebo, and functional magnetic resonance imaging (fMRI) analysis showed that probiotic powder reduced responses to negative emotional stimuli in multiple brain areas compared with placebo. The fMRI is also being used to analyze the relationship between intestinal bacteria and brain function in humans. A study by Tillisch et al. [107] demonstrated that supplementing healthy women (*n* = 36) for 4 weeks with a fermented milk product containing probiotic strains of *Bifidobacterium animalis* subsp. *lactis* CNCM I-2494, *Streptococcus thermophilus* CNCM I-1630, *Lactobacillus bulgaricus* CNCM I-1632 and CNCM I-1519, and *Lactococcus lactis* subsp. *lactis* CNCM I-1631 altered activity of the brain regions that control the central processing of emotion and sensation when compared with that in control groups (unfermented milk product or no intervention).

Several pieces of evidence have accumulated regarding the ability of probiotics to alleviate biological responses under stressful conditions, and several clinical trials have demonstrated that probiotics have beneficial effects by alleviating psychological distress in healthy subjects [102] and by normalizing stress-induced changes in immune properties [108,109] and gastrointestinal symptoms [110]. Takada et al. [111] performed double-blind, randomized, placebo-controlled trials on healthy undergraduate medical students in Japan who were exposed to academic examination stress (*n* = 172). They evaluated the effects of *Lactobacillus casei* Shirota (10^11^ CFUs/day for 8 weeks) on physical symptoms and stress reactivity. Students given placebo showed development and exacerbation of physical symptoms (abdominal and common cold symptoms) accompanied by an increase in stress markers (salivary cortisol levels), but students treated with *Lactobacillus casei* Shirota showed suppression of the onset of these symptoms and significantly suppressed salivary cortisol levels when compared with those given placebo (*p* < 0.05), whose cortisol levels were elevated immediately before the examinations. In a similar study model targeting Japanese medical students (*n* = 69), heat-killed *Lactobacillus gasseri* CP2305 (10^10^ cells/day for 12 weeks) ameliorated chronic-stress-associated responses, including increased salivary cortisol levels and increased expression of stress-responsive microRNAs [112]. In a study by Allen et al. [113], healthy subjects (*n* = 22) were administered placebo for 4 weeks followed by *Bifidobacterium longum* 1714 (1.0 × 10^9^ CFUs/day) for 4 weeks, and were exposed to a cold pressor test to evaluate their stress response. It was demonstrated that increases in salivary cortisol levels and subjective anxiety in response to the cold pressor test were attenuated at post-probiotic than at baseline and post-placebo. They also showed that this probiotic modulated electroencephalographic activity and enhanced cognitive performance.

Exposure to psychological stress causes various symptoms, one of which is sleep disturbance, and the effects of probiotics in helping to maintain sleep quality are now attracting much attention. Heat-killed *Lactobacillus brevis* SBC8803 amplifies diurnal sleep rhythms in mice [114], and this effect has been confirmed in a human study: SBC8803 (25 mg of heat-killed powder/day for 10 days) ameliorated sleep quality in male subjects with slightly challenged sleep (*n* = 17) [115]. *Lactobacillus helveticus* CM4 fermented milk (100 g/day for 3 weeks) improves sleep in healthy elderly subjects (*n* = 30), as evaluated by both wrist actigraphy and questionnaires [116]. *Lactobacillus casei* Shirota (10^11^ CFUs/day for 11 weeks) showed a significant effect in maintaining sleep quality during a period of increasing stress in double-blind, randomized, placebo-controlled trials targeting healthy undergraduate medical students (*n* = 94) [117]; the design of this study was the same as that of the studies mentioned above [111,112]. Overnight electroencephalogram recordings showed that indices of deep sleep were maintained higher in the *Lactobacillus casei* Shirota group than in the placebo group during the intervention (*p* < 0.05), indicating that *Lactobacillus casei* Shirota helped to maintain perceived sleep satisfaction under stressful conditions.

## 5. Possible Mechanisms of Physiological Effects

In this section, we present the latest findings on the dynamics of probiotics in the gastrointestinal tract, and we discuss the mechanisms postulated for the development of physiological effects through probiotic–host cell interaction.

### 5.1. Dynamics in the Small Intestine

After ingestion, probiotics are first exposed to strongly acidic gastric juice and then mixed with bile and pancreatic juice, placing them in a neutral-to-alkaline environment. They therefore reach the ileum and colon after experiencing dramatic pH changes, and here they become symbiotic with the indigenous gut microbiota and host cells. Probiotics are able to meet the requirement to “be alive”, as mentioned in Chapter 1: ingested strains have been recovered from feces in a viable state [118,119]. However, the number of reports showing the behavior of ingested probiotics within the intestinal tract is limited [120,121]. Moreover, the answer to how these organisms travel in the gastrointestinal tract before being excreted in the feces and before exerting their physiological effects remains a black box.

Takada et al. [10] used a technique of small intestinal fluid perfusion by endoscopic retrograde bowel insertion to periodically collect fluids from the terminal ileum of healthy subjects for up to 7 h after ingestion of probiotic (8 × 10^10^ CFUs of *Lactobacillus casei* Shirota or 6 × 10^10^ CFUs of *Bifidobacterium breve* Yakult)-containing fermented milk. Culture of the ileal fluids showed that, despite the dramatic pH changes before reaching the terminal ileum, more than one billion of the ingested probiotics of both strains survived with their colony-forming ability intact. Some other studies also reported that ingested probiotic strains recovered from ileal fluid [120,121,122] and intestinal mucosa [123] with their colony-forming ability intact. As described in Section 2.3, one of the plausible mechanisms for improving constipation symptoms by taking probiotics is the normalization of intestinal function through increased production of SCFAs in association with balancing of the microbiota [34]. The above mentioned results [10,120,121,122,123] support this possibility. It should be noted, however, that the results presented were those observed when ileal fluid and intestinal mucosa was cultured on an enriched agar medium; this method is not sufficient for determining whether the probiotics were actually metabolizing or growing in the small intestine. Therefore, further investigations are needed to clarify whether the probiotics actually produce SCFAs and how they induce the changes in gut microbiota composition in the gastrointestinal tract. Progress of research in this field to elucidate the physiological effects of probiotics in association with changes in the gut microbiota is eagerly anticipated.

**Figure 1:**
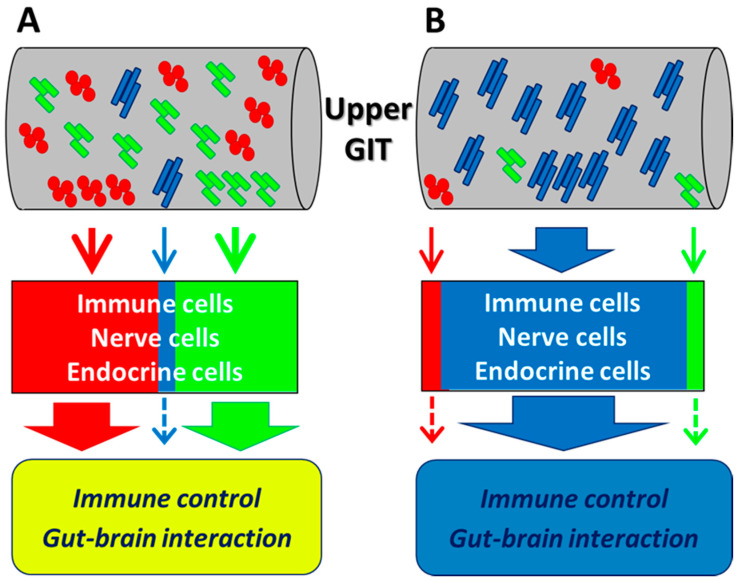
Hypothetical immune control and gut–brain interaction by the intestinal microbiome. (**A**) Immune, nerve, and endocrine cells in the upper gastrointestinal tract (GIT) are continuously stimulated by indigenous bacteria. Even when a small number of probiotics coexist, stimulation by the predominant commensal bacteria affects immune control and the gut–brain interaction. (**B**) During the time period when ingested probiotics occupy the upper GIT, stimulation by the probiotics preferentially contributes to immune control and the gut–brain interaction. Long blue rods: ingested probiotic strain. Red cocci and short green rods: indigenous bacteria.

The above study by Takada et al. [10] revealed some key new findings that will help elucidate the mechanisms of the physiological effects of probiotics. They clearly showed that, after ingestion of fermented milk, the probiotics (i.e., *Lactobacillus casei* Shirota or *Bifidobacterium breve* Yakult) occupied the ileal microbiota for several hours, temporarily representing over 90% of the ileal microbiota in several subjects. In contrast, the relative abundances of major indigenous members of the intestinal microbiota, such as Bacteroidaceae and Lachnospiraceae, decreased. These results reveal the existence of a time period when the small intestinal microbiota is occupied by the ingested probiotics, indicating that there is adequate opportunity for the ingested probiotics to continuously stimulate immune cells, nerve cells, and EECs in the upper gastrointestinal tract. As described in the following sections, stimulation of host cells by probiotics and subsequent signaling have been explained by in vitro experiments and animal studies. However, there has been some skepticism as to whether probiotics can actually interact with host cells in the human gastrointestinal tract, where miscellaneous indigenous bacteria coexist. Normally, immune and endocrine cells in the upper gastrointestinal tract are continuously stimulated by indigenous bacteria and, even when a small number of probiotics coexist, stimulation by the predominant commensal bacteria is thought to affect immune control and the gut–brain interaction (Figure 1A). During the time period when ingested probiotics occupy the microbiota in the upper gastrointestinal tract, stimulation by these probiotics is likely a preferential contributor to these events (Figure 1B). As the dynamics of ingested probiotics in the human gastrointestinal tract become clearer, further progress in this research area should elucidate the behavior of these bacteria within the tract, as well as the mechanism of their physiological effects on the host. The reactions that occur after probiotics encounter the host cells will be discussed in the following sections.

### 5.2. Immune Control

As mentioned for the beneficial effects of probiotics in the Chapter 3, the function of probiotics varies between the strains, and some strains show immune stimulating effects to augment the immune defense against infection and cancer, and others show anti-inflammatory effects to control inflammatory responses, resulting in alleviating the symptoms of IBD. Although the precise mechanisms of their action are not fully known, so far, several active components such as exo-polysaccharides (EPSs), lipoteichoic acids (LTAs), lipopolysaccharides (LPSs) and single-stranded RNA (ssRNA) have been reported [55,74,124,125,126]. EPSs and LTAs are considered to be immune stimulating components. EPS deriving from *Lactobacillus delbrueckii* subsp. *bulgaricus* OLL1073R-1, augmented NK cell activity and induced IFN-γ production through the regulation of phagocytes in mice [55]. LTAs deriving from *Lactobacillus plantarum* L-137 induced IL-12 production in murine splenic DCs [124]. On the other hand, LPSs and ssRNA act to be anti-inflammatory agents. Stimulation of human DCs by specific LPS deriving from *Escherichia coli* Nissle 1917 decreased pro-inflammatory cytokines such as IL-2, TNF-α (tumor necrosis factor alpha) and increased anti-inflammatory cytokines in vitro [74]. ssRNA deriving from *Pediococcus acidilactici* K15, induced IL-10 in the murine DCs [126]. Interestingly, *Lactobacillus casei* Shirota has been reported to have two different active components, rigid cell wall and PSPG-1, that act as an immune stimulating agent and an anti-inflammatory agent, respectively. It has been demonstrated that rigid cell wall of *Lactobacillus casei* Shirota is associated with IL-12 induction in the murine macrophage [127]. The IL-12 production induced by *Lactobacillus casei* Shirota was positively correlated with the enhancement of NK cell activity, and this effect was diminished when treated with anti-IL-12 monoclonal antibody [69]. On the other hand, PSPG-1—a specific cell-wall component of *Lactobacillus casei* Shirota—works to inhibit IL-6 production in LPS-stimulated lamina propria DCs isolated from the murine model of IBD [128]. *Lactobacillus casei* Shirota prevented the development of dextran-sulfate-sodium-induced colon cancer in mice through suppressing IL-6 mRNA expression on DCs in the colonic mucosa, and the effect disappeared in its mutant strain deficient in PSPG-1 [129]. The balance between these two different features of *Lactobacillus casei* Shirota—immune stimulating effects and anti-inflammatory effects—is thought to be flexibly altered depending on the conditions of the host immune system. *Lactobacillus rhamnosus* GG has also been reported to contain two different active components presenting separated features. Gao et al. [130] focused on intestinal epithelial cells rather than phagocytes. Specific surface-layer protein and EPS alleviated LPS-induced IL-6, in contrast CpG-oligodeoxynucleotides increased IL-12 at mRNA level in vitro. Signaling mechanisms of phagocytes or intestinal epithelial cells ingesting or contacting probiotics to present the physiological effects are still not fully understood, and further research is needed.

Recently, Morikawa et al. [131] showed that bacterial sampling by phagocytes has been demonstrated in mice. They found both *Lactobacillus murinus* and *Lactobacillus taiwanensis*, which are abundant in the small intestine of mice under physiological conditions, were incorporated into the small intestinal phagocyte subset. On the other hand, Shima et al. [132] reported that some probiotics affected gene expression of defense/immune functions of the ileum epithelial cells in probiotic monoassociated mice. Thus, it has been demonstrated in mice that bacteria existing within the intestinal tract interact with host phagocytes and epithelial cells. Evidence is awaited as to whether the ingested probiotics actually interact with phagocytes and epithelial cells in the human intestinal tract.

Currently, coronavirus disease 2019 (COVID-19), caused by severe acute respiratory syndrome coronavirus 2 (SARS-CoV-2), is rampant worldwide. The typical symptoms of COVID-19 are fever, dry cough, fatigue, myalgia, dyspnea, and pneumonia, and in severe cases complications such as acute respiratory distress syndrome can occur, ultimately leading to death [133]. One of the causes of this syndrome is a cytokine release syndrome (cytokine storm) predominantly involving IL-6 [134]. Burgueno et al. [135] reported that SARS-CoV-2 was taken up via host ACE2 (angiotensin converting enzyme II) and TMPRSS2 (transmembrane serine protease 2) receptors; these were shown to be expressed in epithelial cells, not only in the lung, but also in the gastrointestinal tract. As mentioned above, certain probiotic strains have been shown to inhibit IL-6 production in the mucosal lamina propria, suggesting that these bacteria could be used to inhibit the progression of cytokine release syndrome. Probiotics may be effective tools for protection against SARS-CoV-2 infection in the intestinal tract, but further study is needed.

### 5.3. Modulation of Brain Function via the Gut–Brain Axis

**Figure 2:**
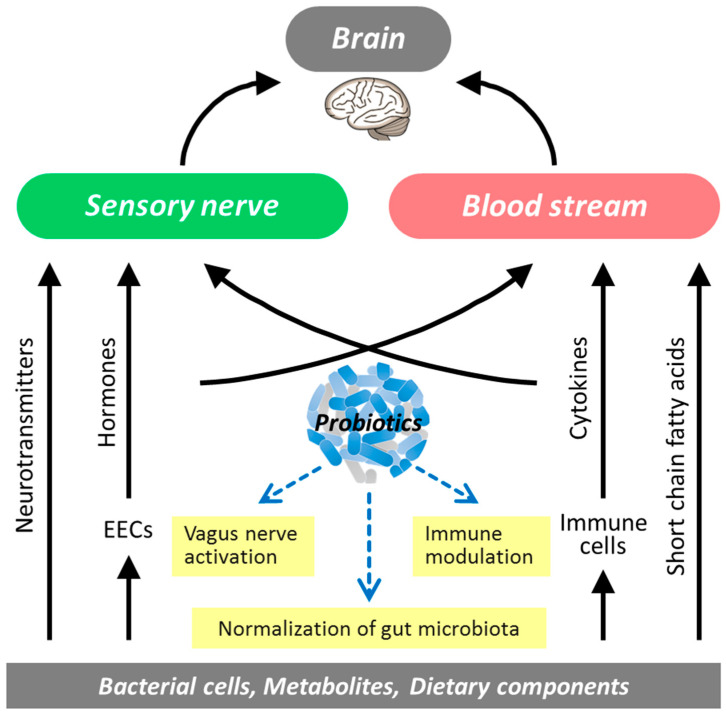
Modulation of the microbiota–gut–brain interaction by probiotics. EECs, enteroendocrine cells.

Accumulating evidence suggests that multiple routes are involved in the action of probiotics on brain function via the gut–brain axis (Figure 2). It has been suggested that microbial cellular components and metabolites of the complex gut microbiota may influence brain functions via blood circulation, humoral pathways, and the immune system as well as via neuronal pathways; differences in microbial diversity and taxonomic composition have been observed between stressed and control individuals [136]. Thus, probiotics beneficial to humans should help to maintain homeostasis of the neuroendocrine and immune systems by preventing disturbance of the gut microbiota. In a human study of medical students under academic examination stress, *Lactobacillus casei* Shirota suppressed stress-related reduction of the α-diversity index of the gut microbiota and lowered Bacteroidaceae abundance [137]. Daily intake of *Lactobacillus gasseri* CP2305 increases α-diversity of the gut microbiota and prevents the stress-induced decline of Bifidobacterium [138,139]. Shifts in gut microbiota diversity have been associated with mental health and psychiatric disorders such as stress, sociability, cognitive function, anxiety, depression, and autism [140]. Thus, the α-diversity index may be a potential marker of the association between the gut microbiota and psychological changes, but meanwhile it must be taken into account that the parameter can be affected by multiple host factors including age and diet. Neuroactive metabolites of the microbiota, such as SCFAs, constitute a route of information flow between the gut and the brain [141]. Butyrate maintains BDNF (brain-derived neurotrophic factor) levels and neurogenesis in the hippocampus and improves depression-like behavior [142,143]. The butyrate-producing bacterium *Clostridium butyricum* MIYAIRI 588 (CBM588) has been effective against depressive symptoms when used in combination with antidepressants [144]. Meanwhile, SCFAs are not always useful and some of them may negatively influence the human organism. There have been several reports that an excessive propionate level may be involved in the progression of autism spectrum disorders [145,146]. Additionally, the mucosal barrier plays an important role in key signaling pathways of microbiota–gut–brain interaction. Deficits in intestinal permeability may underpin the chronic low-grade inflammation observed in disorders such as depression, and probiotics can play a critical role in regulating colonic tight junction integrity [103].

The gut and the brain communicate with each other via multiple pathways, and the major communication route of neuronal pathways is the vagus nerve. Incoming information from the gut via the vagus nerve to the brain is processed in the nucleus tractus solitarius. Recent findings indicate that probiotics can induce the excitability of vagal afferents. An animal study using a water-avoidance stress model confirmed that *Lactobacillus casei* Shirota provokes the activity of the gastric branch of the vagal afferent and suppresses the stress-induced activation of corticotropin releasing factor (CRF)-expressing cells in the paraventricular nucleus of the hypothalamus [111]. This suppresses stress-induced hypersecretion of glucocorticoids through modulation of the reactivity of the HPA axis. Administration of *Lactobacillus brevis* SBC8803 promotes the secretion of serotonin from the small intestine of mice [147]; this is followed by stimulation of activity in the intestinal branch of the vagal afferent and in the stomach’s branch of the vagal efferent. These observations suggest that Lactobacillus probiotic strains modulate stress-induced activation of the HPA axis by acting on the neuroendocrine system. It is widely accepted that stress-induced activation of the HPA axis or the sympathetic nervous system, or both, negatively affects sleep; there is evidence that CRF-initiated stress responses contribute to a decline in sleep quality [148] and that poor sleep is correlated with exaggerated cortisol reactivity [149]. *Lactobacillus casei* Shirota suppresses stress-induced increases in glucocorticoid levels in both a human academic stress model (salivary cortisol) and a rat water-avoidance stress model (plasma corticosterone) [111]; it also suppresses CRF-induced sympathetic activation in rats [150]. These observations have led us to speculate that *Lactobacillus casei* Shirota improves sleep by suppressing the stress induced HPA axis or sympathetic activation, or both.

## 6. Future Perspectives

As described in this review, probiotics are dietary factors that can exert physiological effects on the host through improvement of the gut microbiota and stimulation of host cells. Probiotics are more likely to affect the microbiota of neonates than of adults, as the neonatal microbiome is in the process of formation (see Section 2.1). For established microbiota, such as those of adults, the magnitude of the effect is likely to vary with the types of indigenous microbiota in the population and with the types of strains ingested, the amounts ingested, and the duration of ingestion (see Section 2.2). The mechanisms for increasing or decreasing the abundance of gut microbial families, genera, and species other than ingested probiotics is of great interest in understanding gut microbial ecology. If the physiological effects of probiotics are related to increases or decreases in the production of SCFAs associated with changes in gut microbiota, then simultaneous consumption of prebiotics (which are available specifically for intestinal probiotic strains) should have a stronger effect than consumption of probiotics alone. Because the species and the amounts of SCFAs to be produced in the gastrointestinal tract may vary depending on the symptoms to be improved, a tailor-made combination of probiotics and prebiotics (i.e., synbiotics) may be required for each individual. Whether probiotics that reach the small and large intestines produce SCFAs such as lactate and acetate in situ, and how these SCFAs affect different types of indigenous bacteria, are issues that need to be clarified to reveal the mechanisms behind the physiological effects of probiotics.

As described in Section 5.2 and Section 5.3, the mechanism by which probiotics exert physiological effects on the host by interacting directly with host cells has also been discussed. In vitro and animal studies have shown that probiotics stimulate immune cells, nerve cells, and endocrine cells. Health benefits such as infection defense, immune control, cancer prevention, stress relief, and improved sleep quality are probably exerted through such mechanisms. Recent research advances have revealed the crucial function of neuroimmune interactions in tissue homeostasis, and the newly discovered pathway by which sensory information on the gut microenvironment is transmitted to the brain via vagal afferents, and the reflex action modulates gut immune homeostasis, is being clarified. We need to elucidate how probiotics are involved in the interactions between the immune, nerve, and endocrine systems to exert physiological effects on the host. Recent human studies have shown that probiotics have adequate opportunity to continuously stimulate host cells in the small intestine over a period of time. The next challenge is to make the connection between occupation of the small intestine by ingested probiotics and their stimulation of host cells; in other words, we need to prove that probiotics actually contact and signal host cells in the human gastrointestinal tract.

Finally, we would like to mention the safety issue of probiotics. As Hills et al. [151] stipulated in a consensus statement on the scope and appropriate use of the term probiotic, which was released by The International Scientific Association for Probiotics and Prebiotics, “all probiotics must be safe for their intended use”. Many species of lactic acid bacteria, bifidobacteria and yeasts have been judged as safe for use in foods and supplements in the bulletin of the International Dairy Federation, because they belong to genera and species with a documented history of safe use [152]. Meanwhile the evaluation of risk factors at strain level, especially the absence of acquired antimicrobial resistance genes or known virulence factors such as toxins, invasion, and adhesion factors, is required. A recent study by Suez et al. [153] showed that probiotics consumption following antibiotic exposure caused long-term colonization of probiotics in the human gut, which was associated with significant delay of post-antibiotic gut microbiota reconstitution. Providing consumers with probiotic foods and supplements that are safe is a basic responsibility of manufacturers, and the safety of probiotics is an important issue that should continue to be properly discussed in the future.

## Figures and Tables

**Table 1:** Effects of probiotics on upper respiratory tract infections (URTIs) in human studies.

References	Probiotics (CFUs/Day)	Target Subjects	Result
[56]	*Lactobacillus casei* DN-114001 (2.0 × 10^10^)	Children (3–6 years) (*n* = 638)	Incidence of common infectious diseases decreased by ingestion of probiotic drink.
[55]	*Lactobacillus delbrueckii* subsp. *bulgaricus* OLL1073R-1 (2.5 × 10^10^)	Elderly (*n* = 142)	Risk of catching a cold decreased by intake of yogurt.
[57]	*Lactobacillus casei* Shirota (1.3 × 10^10^)	Athletes (*n* = 84)	Incidence of URTI was reduced by intake of probiotic drink.
[58]	*Lactobacillus casei* Shirota (4.0 × 10^10^)	Elderly (*n* = 154)	Probiotic treatment reduced the duration of acute URTIs.
[59]	*Lactobacillus brevis* KB290 (6.0 × 10^9^)	Schoolchildren (6–12 years) (*n* = 1089)	Incidence of influenza was reduced by intake of probiotic drink.
[61]	*Bifidobacterium animalis* subsp. *lactis* BB-12 (1.0 × 10^10^)	Infants (1 month) (*n* = 109)	Incidence of URTI was reduced by probiotic treatment.
[60]	Sachet: *Bifidobacterium bifidum* W23, *Bifidobacterium lactis* W51, *Enterococcus faecium* W54, *Lactobacillus acidophilus* W22, *Lactobacillus brevis* W63 and *Lactococcus lactis* W58 (1.0 × 10^10^)	Athletes (*n* = 33)	Incidence of URTI was reduced by probiotic treatment.
[63]	*Lactobacillus casei* Shirota (1.0 × 10^11^)	Stress-burdened office workers (*n* = 96)	Incidence of URTI was reduced by probiotic drink.
[62]	*Lactobacillus paracasei* N1115 (1.0 × 10^10^)	Healthy elderly over 45 years (*n* = 205)	The incidence of acute URTI was reduced by probiotic treatment.
[64]	*Lactobacillus plantarum* DR7 (1.0 × 10^9^)	Adults (*n* = 109)	Probiotic treatment reduced the duration of nasal symptoms and the frequency of URTI.
